# Supplementary material for: Helicobacter pylori initiates successful gastric colonization by utilizing L-lactate to promote complement resistance
Source: Nat Commun. 2023 Mar 27;14:1695. doi: 10.1038/s41467-023-37160-1 (PMC10042806; doi:10.1038/s41467-023-37160-1)
Supplement: Supplementary file 1 — Supplementary Information [file 41467_2023_37160_MOESM1_ESM.pdf]

Supplemental Table 1 Strains used for this study

Strain	Genotype or description	Reference and/or source(s)
PMSS1	WT strain	Arnold et al., 2011
PMSS1 *ΔlctP*	*Δhp0140-0141::cat*	This study (KO1710)
PMSS1 pTM115	GFP^{+} PMSS1 WT	This study (KO1568)
PMSS1 *ΔlctP* pTM115	GFP^{+} PMSS1 *ΔlctP*	This study (KO1711)

Supplemental Table 2 Primers used to delete *lctP* coding genes

Primer name	Sequence
5’-*lctP*_fwd	TCCTGTGCATGAACCGCTGGGCAA
5’-*lctP*_rev	CTATATCATAAGAAAAAACTAATTTTAAGTAGCATTTAATATTAAG TCAAATTTAATGG
*cat*_fwd	AGTTTTTTCTTATGATATAGTGGATAGATTTATGATATAATGAGTT ATCAACAAATCG
*cat*_rev	CAAAATGGCCTCCGCAGGACGCACTACTC
lctP-3’_fwd	GTCCTGCGGAGGCCATTTTGGATCTGGAAG
lctP-3’_rev	AAAAACACACGCTTAAGAAAAAAC

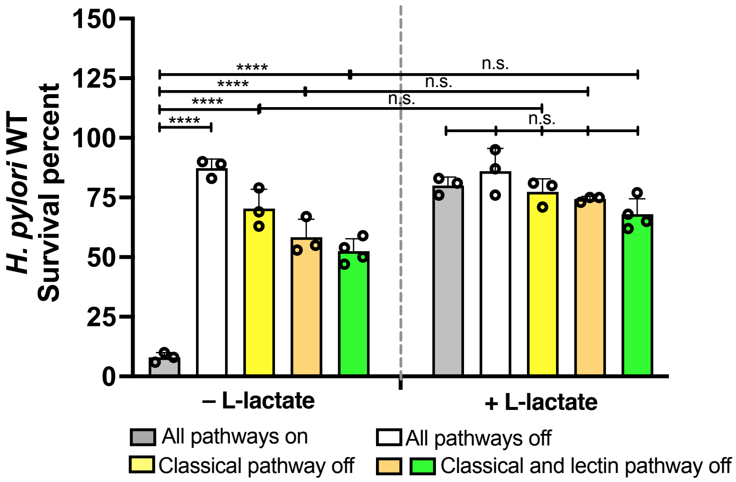

Supplemental Figure 1 L-lactate protects *H. pylori* from classical complement pathway mediated elimination

Overnight cultures of *H. pylori* PMSS1 WT, with or without L-lactate supplementation, were treated by inactive and active human serum at 30% final concentration for one hour, and then viable bacterial CFUs were determined by plating. The survival percent was determined as described in Fig. 2. In the panel, *H. pylori* cultures were treated by NHS only (grey bar), EGTA-conditioned NHS (white bar), C1q-depleted human serum (yellow bar), C2-depleted human serum (orange bar), or EGTA + Mg^{2+} (green bar) as in Fig. 3d. All tests were applied with 30% serum. The results were presented as survival percent ± standard deviation (SD), and were derived from triplicate biological samples with a triplicate of each given sample. The *p*-values were obtained by one-way ANOVA with Tukey's multiple comparisons test. The significance is indicated as **** ($p < 0.0001$), or n.s. (not significant). Source data and exact *p*-values are provided in the Source Data file.

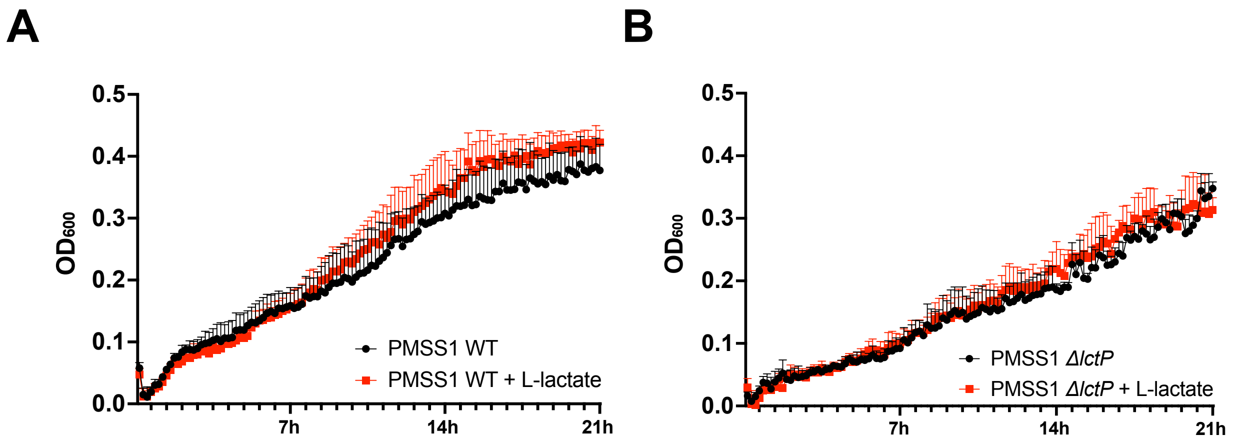

Supplemental Figure 2 LctP is required for growth advantage acquirement

Growth of *H. pylori* PMSS1 wild type (A), the *ΔlctP* mutant (B) were cultured in BB10 medium with (red dots) or without (black dots) L-lactate supplementation (10 mM of final concentration). The cell growth was monitored by measuring optical density at 600 nm (OD_{600}). The data points and error represent the mean ± standard deviation (SD) of three independent experiments.

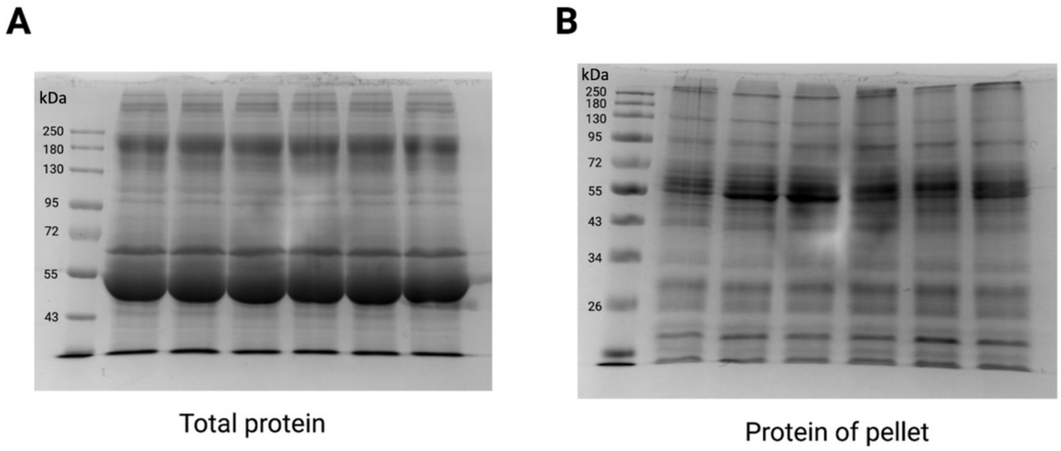

Supplemental Figure 3 equal amount of pelleted *H. pylori* was loaded for examination

H. pylori PMSS1 WT was grown ± L-lactate and then treated by 10% NHS for the indicated time periods. Each sample was split, (A) with one sample treated by sample buffer only (total protein), or (B) centrifuged at 3,000g for 3 min before adding sample buffer without BME to equal amounts as in (A) (protein of pellet). Equal amounts of protein samples were loaded as for Fig. 4c. Protein samples were run on 7-10% SDS PAGE and stained by Coomassie Blue. The result (A, B) is representative of three independent experiments with triplicate biological samples.

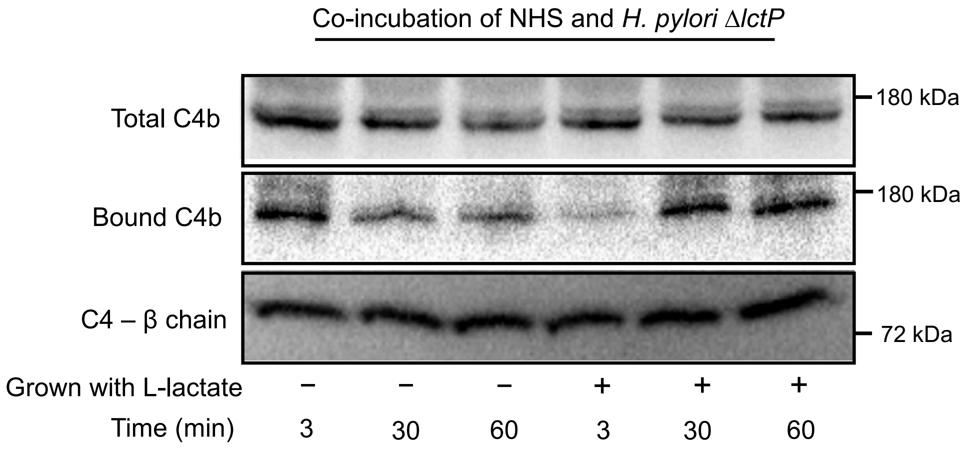

Supplemental Figure 4 Bound C4b was stably bound to Δ*lctP* surface

H. pylori PMSS1 *ΔlctP* mutant was grown ± L-lactate and then treated by 10% NHS for the indicated time periods. Protein samples were prepared and blotted as for WT *H. pylori* showed in Fig. 4c. The result is representative of three independent experiments with triplicate biological samples.

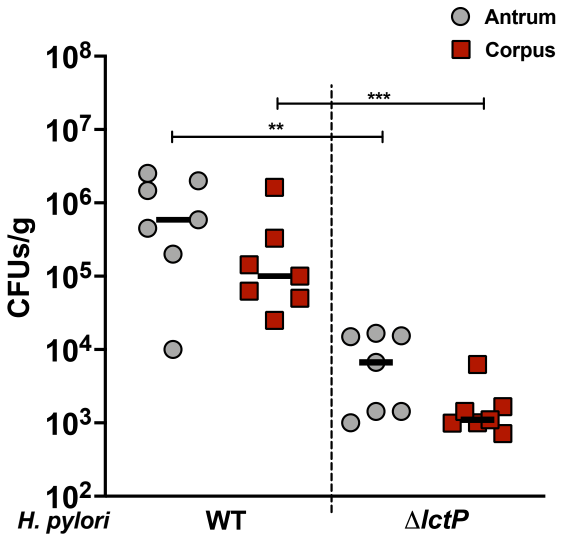

Supplemental Figure 5 Δ*lctP* mutant is deficient to colonize C57BL/6N mice.

C57BL/6N mice (n = 7 per group) were infected by oral gavage with GFP⁺ *H. pylori* PMSS1 WT or *ΔlctP* mutant for two weeks. Stomachs were isolated from infected mice and separated into corpus and antrum pieces. A piece tissue of each region was homogenized and plated to obtain the total colony forming units (CFUs). Total bacterial number from antrum (gray) and corpus (red) of infected C57Bl6/N mice normalized to tissue weight (CFUs/g). Statistical analysis were performed using two-tailed Mann–Whitney *U*-test. The significance was indicated as ** ($p < 0.01$), *** ($p < 0.001$). Source data and exact *p*-values are provided in the Source Data file.
