# Supplementary material for: Helicobacter pylori initiates successful gastric colonization by utilizing L-lactate to promote complement resistance
Source: Nat Commun. 2023 Mar 27;14:1695. doi: 10.1038/s41467-023-37160-1 (PMC10042806; doi:10.1038/s41467-023-37160-1)
Supplement: Supplementary file 3 — Reporting Summary [file 41467_2023_37160_MOESM3_ESM.pdf]

nature portfolio

Corresponding author(s): Karen M. Ottemann

Last updated by author(s): Feb 28, 2023

Reporting Summary

Nature Portfolio wishes to improve the reproducibility of the work that we publish. This form provides structure for consistency and transparency in reporting. For further information on Nature Portfolio policies, see our Editorial Policies and the Editorial Policy Checklist.

Statistics

For all statistical analyses, confirm that the following items are present in the figure legend, table legend, main text, or Methods section.

n/a	Confirmed	
☐	☒	The exact sample size (*n*) for each experimental group/condition, given as a discrete number and unit of measurement
☐	☒	A statement on whether measurements were taken from distinct samples or whether the same sample was measured repeatedly
☐	☒	The statistical test(s) used AND whether they are one- or two-sided<br>*Only common tests should be described solely by name; describe more complex techniques in the Methods section.*
☒	☐	A description of all covariates tested
☐	☒	A description of any assumptions or corrections, such as tests of normality and adjustment for multiple comparisons
☐	☒	A full description of the statistical parameters including central tendency (e.g. means) or other basic estimates (e.g. regression coefficient) AND variation (e.g. standard deviation) or associated estimates of uncertainty (e.g. confidence intervals)
☐	☒	For null hypothesis testing, the test statistic (e.g. *F*, *t*, *r*) with confidence intervals, effect sizes, degrees of freedom and *P* value noted<br>*Give P values as exact values whenever suitable.*
☒	☐	For Bayesian analysis, information on the choice of priors and Markov chain Monte Carlo settings
☒	☐	For hierarchical and complex designs, identification of the appropriate level for tests and full reporting of outcomes
☒	☐	Estimates of effect sizes (e.g. Cohen's *d*, Pearson's *r*), indicating how they were calculated

Our web collection on statistics for biologists contains articles on many of the points above.

Software and code

Policy information about availability of computer code

Data collection	Chemidoc MP imaging system, used for western blot gels imaging.
Data analysis	GraphPad Prism version 9, used for for all statistical analysis in this study.<br>Chemidoc MP imaging system, used for gel intensity analysis

For manuscripts utilizing custom algorithms or software that are central to the research but not yet described in published literature, software must be made available to editors and reviewers. We strongly encourage code deposition in a community repository (e.g. GitHub). See the Nature Portfolio guidelines for submitting code & software for further information.

Data

Policy information about availability of data

All manuscripts must include a data availability statement. This statement should provide the following information, where applicable:

- Accession codes, unique identifiers, or web links for publicly available datasets
- A description of any restrictions on data availability
- For clinical datasets or third party data, please ensure that the statement adheres to our policy

The authors declare that the data supporting the findings of this study are available within the paper and its Supplementary Information files. Source data are provided with this paper.

Human research participants

Policy information about studies involving human research participants and Sex and Gender in Research.

Reporting on sex and gender	Not applicably
Population characteristics	Not applicably
Recruitment	Not applicably
Ethics oversight	Not applicably

Note that full information on the approval of the study protocol must also be provided in the manuscript.

Field-specific reporting

Please select the one below that is the best fit for your research. If you are not sure, read the appropriate sections before making your selection.

☒ Life sciences ☐ Behavioural & social sciences ☐ Ecological, evolutionary & environmental sciences

For a reference copy of the document with all sections, see nature.com/documents/nr-reporting-summary-flat.pdf

Life sciences study design

All studies must disclose on these points even when the disclosure is negative.

Sample size	A statement describing sample size determination was added to the mouse description: Sample sizes were chosen based on previous H. pylori mouse experiments.
Data exclusions	The only data that was excluded was one mouse from the C3-/- 1-year infection with lctP mutant. This mouse had very low levels of colonization, below the 25% of the average of the other 4 mice in the group and was excluded.
Replication	Replicates are shown in the figures for the serum killing, mouse assays, and C4 western blots.
Randomization	Randomization was states in the Statistical analysis and Reproducibility: Allocation of mice was random in all in vivo experiments, taken from littermates.
Blinding	The investigators were not blinded to allocation during the experiments and out assessment. All experiments required known introduced H. pylori strains and mouse strains. Therefore, it was not possible to blind the investigator for those experiments.

Reporting for specific materials, systems and methods

We require information from authors about some types of materials, experimental systems and methods used in many studies. Here, indicate whether each material, system or method listed is relevant to your study. If you are not sure if a list item applies to your research, read the appropriate section before selecting a response.

Materials & experimental systems

n/a	Involved in the study	
☐	☒	Antibodies
☒	☐	Eukaryotic cell lines
☒	☐	Palaeontology and archaeology
☐	☒	Animals and other organisms
☒	☐	Clinical data
☒	☐	Dual use research of concern

Methods

n/a	Involved in the study	
☒	☐	ChIP-seq
☒	☐	Flow cytometry
☒	☐	MRI-based neuroimaging

Antibodies

Antibodies used	Rabbit anti-human C4 antibody (mAB #60059) (Cell Signaling)
Validation	More validation information of this antibody and the publications that cited this antibody can be found: https://www.cellsignal.com/

Validation	products/primary-antibodies/complement-c4-a-c4-b-e6j7w-rabbit-mab/60059.

Animals and other research organisms

Policy information about studies involving animals; ARRIVE guidelines recommended for reporting animal research, and Sex and Gender in Research

Laboratory animals	Mus musculus C57BL/6J; Mus musculus C57BL/6N; Mus musculus B6;129S4-C3<tm1Crr>/J mice (C3-/-).<br>All mice were between 7-9 weeks old at the time of H. pylori infection.<br>C57BL/6J WT and C3-/- littermates were used for H. pylori infection.<br>Numbers of mice are provided within each figure legend.<br>Mice were housed under 1:1 (light:dark) light cycles, at 21-23 degrees (C), and 50-60% humidity, These conditions are checked and maintained by vivarium staff daily.
Wild animals	No wild animals
Reporting on sex	included in methods
Field-collected samples	No field collected samples.
Ethics oversight	Animal protocols approved by UC Santa Cruz IACUC

Note that full information on the approval of the study protocol must also be provided in the manuscript.
